# 2009 A(H1N1) Seroconversion Rates and Risk Factors among the General Population in Vientiane Capital, Laos

**DOI:** 10.1371/journal.pone.0061909

**Published:** 2013-04-18

**Authors:** Alexia Kieffer, Phimpha Paboriboune, Pascal Crépey, Bruno Flaissier, Vimalay Souvong, Nicolas Steenkeste, Nicolas Salez, François-Xavier Babin, Christophe Longuet, Fabrice Carrat, Antoine Flahault, Xavier de Lamballerie

**Affiliations:** 1 UMR 190, Aix-Marseille Université - IRD - EHESP, Marseille, France; 2 Centre d'Infectiologie Christophe Mérieux du Laos, Vientiane Capital, Laos; 3 EHESP Rennes, Sorbonne Paris Cité, Paris, France; 4 Fondation Mérieux, Lyon, France; 5 UMR-S 707, INSERM UPMC, Paris, France; The Australian National University, Australia

## Abstract

**Objective:**

To assess 2009 A(H1N1) seroconversion rates and their determinants within an unvaccinated population in Vientiane Capital, Laos.

**Methods:**

CoPanFlu Laos, a general population cohort of 807 households and 4,072 participants was established in March 2010. Sociodemographic data, epidemiological data, and capillary blood samples were collected from all the household members in March, and again in October 2010, in order to assess the level of antibodies to 2009 A(H1N1) with the haemagglutination inhibition assay. 2009 A(H1N1) seroconversion was defined as a fourfold or greater increase in titre between inclusion and follow-up. Determinants for pandemic influenza infection were studied using the generalized estimating equations model, taking household clustering into account.

**Results:**

Between March and November 2010, 3,524 paired sera were tested. Prior to the pandemic, our cohort was almost completely vaccine-naive for seasonal influenza. The overall seroconversion rate among nonvaccinated individuals (*n* = 2,810) was 14.3% (95%CI [13.0, 15.6]), with the highest rate for participants under 20 yo (19.8%, 95%CI [17.4, 22.4]) and the lowest rate for participants over 60 yo (6.5%, 95%CI [3.7, 10.4]). Participants with lower baseline titres had significantly higher infection rates, with a dose-effect relationship. Odds ratios (ORs) ranged from 76.5 (95%CI [27.1, 215.8]), for those with a titre at inclusion of 1∶10, to 8.1 (95%CI [3.3, 20.4]), for those with a titre of 1∶40. Having another household member with a titre ≥1∶80 was associated with a higher likelihood of immunity (OR = 3.3, 95%CI [2.8, 3.9]).

**Conclusion:**

The determinants and age distribution for seroconversion within a vaccine-naive population were similar to those found in developed countries. This pandemic was characterized by strong epidemiological determinants, regardless of geographical zone and level of development. Moreover, we detected pre-existing cross-reacting antibodies in participants over 60 yo, which could not have originated from former multiple vaccination as has been suggested elsewhere.

## Introduction

Epidemiological information on influenza in tropical countries traditionally remains limited, compared with temperate countries [1]. However, since 2003, the situation has changed dramatically in Asia-Pacific, especially Southeast Asia, as this region is regarded as a potential epicentre of the next pandemic [2]. In Laos, in response to concerns over avian influenza A(H5N1), national governance on pandemic preparedness has been developed [2], and laboratory-based influenza surveillance has been implemented in the capital city of Vientiane [3].

This surveillance system provided useful information about the spread of 2009 influenza A(H1N1) pandemic virus (2009 A(H1N1)). The first case in the Lao People's Democratic Republic (PDR) was confirmed on 16 June 2009, by the National Centre for Laboratory and Epidemiology (NCLE) in Vientiane Capital [4]. Between 28 April 2009 and 1 June 2010, the NCLE confirmed 663 cases of influenza, including 335 cases that tested positive for pandemic virus (50.5%), the remaining cases (49.5%) being variations of seasonal influenza [5], mostly A(H3) virus (NCLE data, unpublished). Pandemic cases were reported in 13 of the country's 17 provinces, but were mainly recorded in Vientiane Capital and Vientiane Province, in the south and centre of the country. Two deaths associated with pandemic virus infection were identified. Since July 2010, biological investigations of pandemic influenza virus, and epidemiological reporting have been limited. However, data collected from neighbouring countries in Southeast Asia suggest that the pandemic virus continues to circulate, and that the number of confirmed cases does not reflect the true number of infections [5].

In order to investigate the burden of 2009 A(H1N1) influenza within the general population of Vientiane Capital, we set out to assess 2009 A(H1N1) seroconversion rates and their determinants within a cohort of households, implementing the first ever sero-epidemiological investigation of influenza in Laos. In this article, we report the results of the first two phases of this programme in a population that was virtually vaccine-naïve for influenza, insofar as influenza vaccine was not routinely available in the country prior to the pandemic [3].

## Materials and Methods

### Ethics Statement

Approval was obtained from the Lao National Ethics Committee for Health Research in Lao PDR (266/NECHR; 23 October 2009). All participants provided their written informed consent. Parents or legal guardians were asked to sign on behalf of children under 17 yo.

### Study design and population

The Cohort for Pandemic Influenza in Laos (CoPanFlu-Laos) was established as part of the CoPanFlu International Consortium research programme. This programme was implemented in the wake of the influenza pandemic alert in April 2009, in order to study the epidemiological, environmental and virological determinants of influenza infection in different regions of the world (*i.e*., metropolitan France [6], the Indian Ocean [7], Africa [8], South America and Southeast Asia).

#### Cohort study

The Laotian study was conducted within the administrative boundaries of the Vientiane metropolitan area. The household cohort was established on the basis of a pre-existing sample constituted for the "Urbanization, Governance and Spatial Disparities of Health in Vientiane” research programme [9]–[11]. Households were recruited in 27 neighbourhoods, distributed across three zones: (*i*) the central zone; (*ii*) the inner urbanized belt and (*iii*) the outer urbanized belt, in order to reflect the variability of Vientiane's overall population [10].

Households were only included in the study if all the members agreed to take part (consent for children aged 16 or under had to be provided by their parents). A household was defined as a group of individuals living on the same plot of land (one or more houses) or in the same building (apartment) and sharing their meals.

***The inclusion visit (March–April 2010)*** was organized as follows: a team of interviewers visited the household at an appointed date in order to present the project, collect the written consent forms from each household member, identify a “referent” household member, collect inclusion information and draw the blood samples. If any household member refused to take part, the household was not included in the study. The questionnaires collected information on the participants' demographics, work environment, general health status, history of influenza-like illnesses (ILIs) and other chronic and acute diseases, their history of vaccination, living conditions and household environment.

A capillary blood sample was systematically collected from each member of the household with a single-use automatic lancing device, and taken to the Centre d'Infectiologie Christophe Mérieux of Lao PDR (CICML) for centrifugation, aliquoting and storage at −80 °C.

***The follow-up phase (October–November 2010)***, consisted of a systematic visit to each household in order to take a second capillary blood sample from each member, using the same methodology as at the inclusion visit, and to update the demographic, environmental and clinical data.

**Figure 1:**
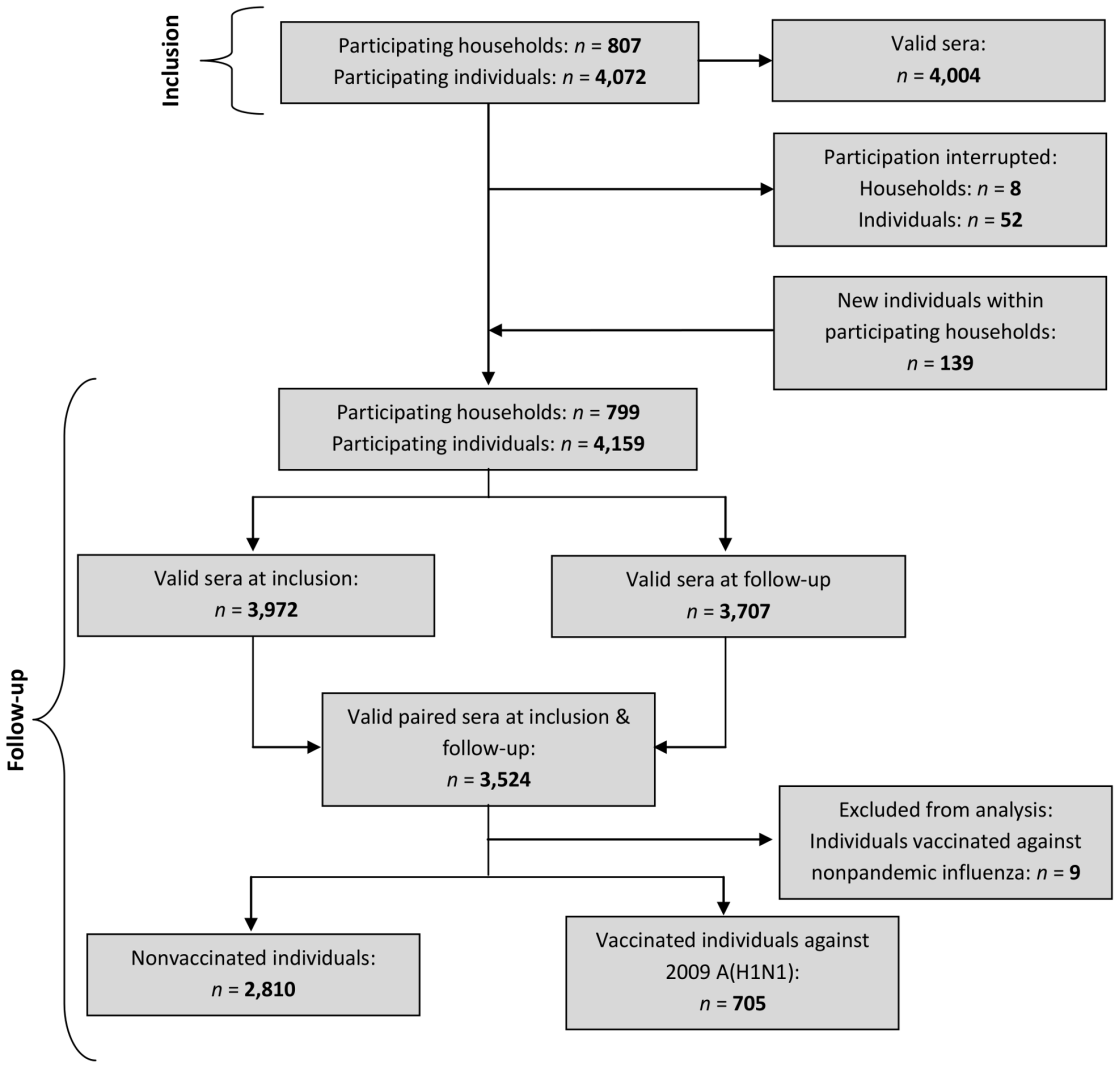
Flowchart of inclusion and follow-up phases.

See Figure 1 for the study's flowchart at inclusion and follow-up.

#### Prepandemic data

The Lao Red Cross (LRC) blood bank provided 1,000 plasma samples that had been collected in 2008 from donors aged 17–58 years living in Vientiane Capital. These samples were used to investigate the presence of antibodies to 2009 A(H1N1) in the population before the pandemic. These samples came from blood donations that were suitable for transfusion (*i.e.*, testing negative for the human immunodeficiency virus, the hepatitis B and C viruses, and syphilis). Information about age and sex was available for these samples.

#### Expected number of participants and justification

Based on the hypothesis of a 30% attack rate, we calculated that a follow-up of 731 households in Vientiane Capital, Lao PDR (estimated to total around 3,000 persons) would ensure statistical power above 80% and allow us to identify the risk factors for exposure levels of 10–90% and a relative risk greater than 1.4.

### Laboratory methods

A standard haemagglutination inhibition (HI) technique was performed to detect and quantify antibodies to the 2009 A(H1N1) virus, as detailed elsewhere [7], [8], [12]. Twofold serum dilutions (1∶10–1∶1280) were tested using a fully automated protocol. The HI titre was determined as the last dilution providing complete inhibition of haemagglutination. All the analyses were performed in the presence of a serum agglutinating activity control and the same negative and positive controls, the latter including sera with titres ranging from 1∶10 to 1∶1280.

### Data analysis and statistics

All the serological data were analyzed according to the participants' pandemic virus vaccination status. Nine participants who were vaccinated against nonpandemic virus (*i.e*., seasonal prepandemic influenza strains) between March and October 2010 were excluded from the analysis. These participants belonged to six different households (two in the inner urbanized belt, four in the outer rural belt). None of the participants seroconverted for pandemic influenza. Six of them were women, and their median age was 44.5 yo [range: 22.4–58.6].

Seroprevalence of 2009 A(H1N1) antibodies at a given HI titre was calculated as the percentage of samples with an antibody titre equal to or above the threshold titre under consideration. Seroconversion was defined as a fourfold or greater increase in antibody titres.

In order to compare seroprevalence between blood donors sampled in 2008 and the participants included in the cohort in 2010, individuals were matched for age (±2 years) and sex using the greedy algorithm, with the %GMATCH macro in SAS® version 9.1.3 (SAS Institute Inc., Cary, NC). Differences in seroprevalence between matched individuals were assessed with the McNemar chi-square test for matched pairs.

GMTs were calculated by assigning a titre of 5 to specimens in which no HI antibody was detected. The following log transformation was used: log HI titre = log_2_ (HI titre/5). The maximum likelihood estimate of the GMT of truncated HI titres and its 95% confidence interval were obtained by means of a statistical technique based on survival analysis, using PROC LIFEREG in SAS® version 9.1.3 (SAS Institute Inc., Cary, NC), avoiding underestimation for censored observations [13].

A generalized estimating equations (GEE) model was used to identify factors associated with seroconversion, taking household clustering into account. This analysis included 2,810 nonvaccinated participants (720 households) for whom full inclusion and follow-up information was available regarding age, serology results and selected risk factors. A univariate screening analysis was run on all the variables. Variables associated with seroconversion with a *p* value ≤0.2 were included in the multivariate regression model.

## Results

### Characteristics of the sample

Between 11 March 2010 and 11 April 2010, we recruited 4,072 individuals belonging to 807 households. The main characteristics of the cohort at inclusion are set out in Table 1 for households and Table 2 for participants. Participants for whom we were able to collect serum samples at inclusion and follow-up (*n* = 3,524) had a median age of 28.1 years (range: 0.7, 90.6) and 54% (*n* = 1,902) were women. In all, 207 households (33.1%) were located in the central zone of Vientiane Capital, 216 (26.8%) in the inner urbanized belt and 324 (40.1%) in the outer urban belt. A total of 23% of participants (*n* = 714) were vaccinated against influenza in 2010, of whom 98.7% (*n* = 705) were vaccinated against pandemic influenza (Influenza A (H1N1) 2009 Monovalent Vaccine, CSL Limited, nonadjuvanted).

**Table 1:** Characteristics of the recruited households at inclusion.

Household description (*N* = 807)	
Level of urbanization*	
- Central zone	3.1%)
- Inner urbanized belt	216 (26.8%)
- Outer urbanized belt	324 (40.1%)
Number of individuals per household (mean ± *SD*)	5.0±2.21
Number of children per household (mean ± *SD*)	1.35±1.19
Number of rooms per household (mean ± *SD*)	2.9±1.33
Ethnic origin: Lao Lum	789 (97.8%)

* according to Vallée *et al.*, Emerging themes in epidemiology. 2007.

**Table 2:** Characteristics of the recruited individuals at inclusion.

CoPanFlu cohort (*N* = 4,072)	
Median age (range)	26.5 [0.1–94.5]
Female	2,183 (53.6%)
Able to read and write (participants≥16 yo)	2,800 (93.6%)
Students	1,348 (33.1%)
In employment	1,769 (43.4%)
Chronic disease	473 (11.6%)
Main chronic diseases reported	
- High blood pressure	210 (5.2%)
- Gastralgia	158 (3.9%)
- Diabetes	74 (1.8%)
- Asthma	37 (0.9%)
History of acute diseases	
- Dengue	385 (9.5%)
- Malaria	314 (7.7%)
- Pneumonia	73 (1.8%)
- Tuberculosis	5 (0.1%)
Body mass index (mean ± *SD*)	21.7±5.0
History of influenza over past three years	165 (4.1%)
Influenza vaccination in 2009	22 (0.5%)
Pneumococcal vaccination	7 (0.2%)
Current smoker	481 (11.8%)

All the information (except BMI) was provided by the participants during the inclusion interview.

### Seroprevalence at inclusion in nonvaccinated participants (n = 2,810)

**Figure 2:**
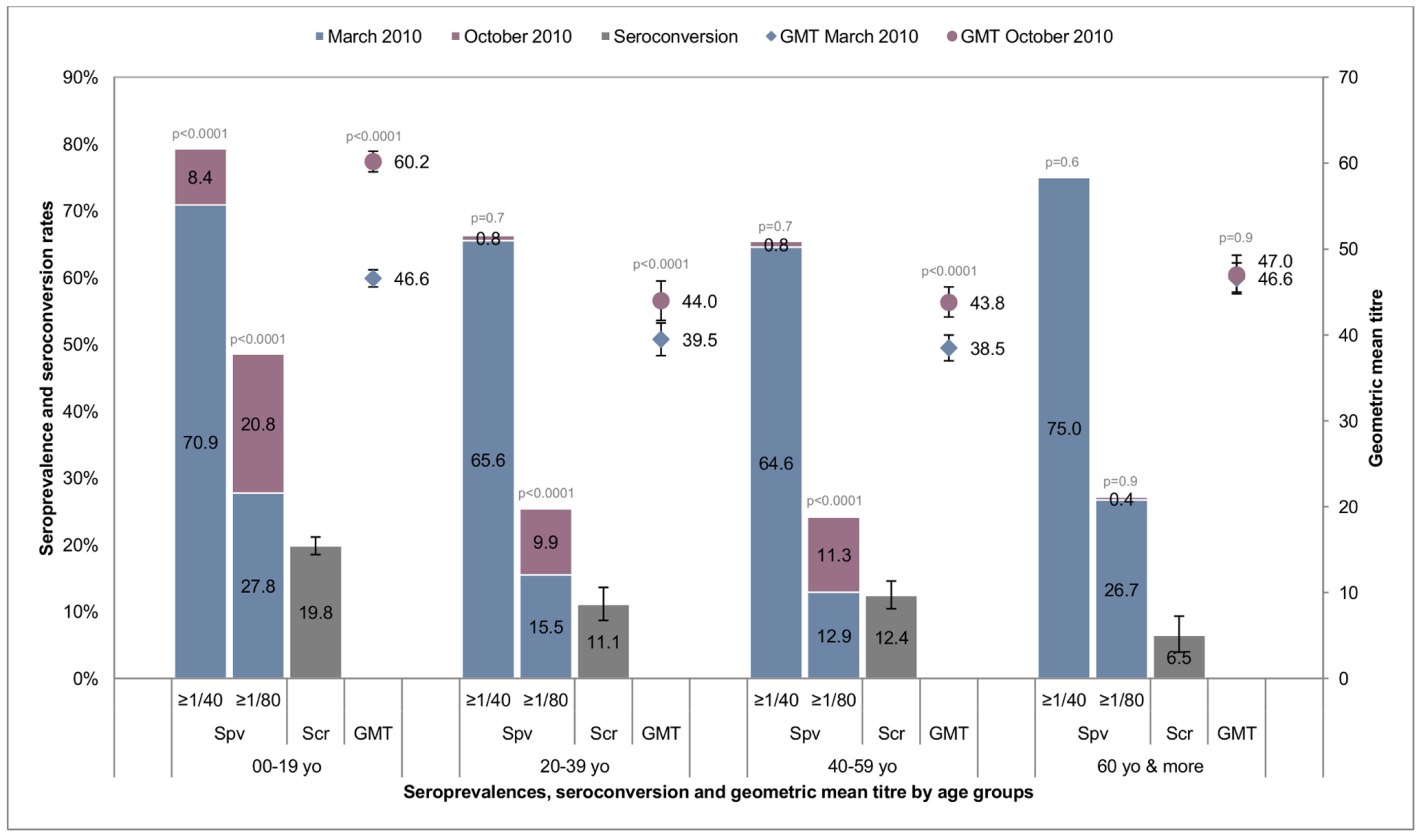
Seroprevalence, seroconversion rates and GMT in March and October 2010 in 2,810 nonvaccinated individuals. Error bars represent confidence intervals. Spv: seroprevalence. Scr: seroconversion.≥1∶40: Seroprevalence analyzed with a cutoff≥1∶40.≥1∶80: Seroprevalence analyzed with a cutoff≥1∶80. p value calculated using the McNemar chi-square for seroprevalence matched pairs. p value calculated using the Wilcoxon signed rank test for GMT matched pairs.

Table 3 and Figure 2 show the results of the HI analyses for each age group. For thresholds of either 1∶40 (Table 3a), or 1∶80 (Table 3b), the distribution of seroprevalence at inclusion followed a U curve, with the highest prevalence for those below 20 yo and over 60 yo. GMT values followed a similar pattern (Table 3c, Fig. 2).

**Table 3:** Seroprevalence and GMT in March and October 2010 in 2,810 nonvaccinated participants.

Table 3a					
HI≥1∶40					
	N	March 2010	October 2010	Δ≥1∶40^§^	*p* value*
**All age groups**	2,810	68.1% [66.3–69.8]	71.5% [69.8–73.1]	3.4	0.0018
**00–19 yo**	1,034	70.9% [68.0–73.6]	79.3% [76.7–81.7]	8.4	<0.0001
**20–39 yo**	929	65.6% [62.4–68.6]	66.3% [63.2–69.4]	0.7	0.7
**40–59 yo**	613	64.6% [60.7–68.4]	65.4% [61.5–69.2]	0.8	0.7
**60+yo**	232	75.0% [68.9–80.4]	72.8% [66.6–78.5]	−2.2	0.6

Table 3b					
HI≥1∶80					
	N	March 2010	October 2010	Δ≥1∶80^§^	*p* value*
**All age groups**	2,810	20.4% [18.9–21.9]	33.8% [32.1–35.6]	13.4	<0.0001
**00–19 yo**	1,034	27.8% [25.1–30.6]	48.6% [45.5–51.6]	20.8	<0.0001
**20–39 yo**	929	15.5% [13.2–18.0]	25.4% [22.6–28.3]	9.9	<0.0001
**40–59 yo**	613	12.9% [10.3–15.8]	24.1% [20.8–27.7]	11.2	<0.0001
**60+yo**	232	26.7% [21.2–32.9]	27.2% [21.5–33.4]	0.5	0.9

Table 3c					
GMT					
	N	March 2010	October 2010	Δ GMT^§^	*p* value**
**All age groups**	2,810	42.4 [41.4–43.4]	49.9 [48.7–51.1]	7.5	<0.0001
**00–19 yo**	1,034	46.6 [44.7–48.5]	60.2 [57.9–62.5]	13.6	<0.0001
**20–39 yo**	929	39.5 [38.0–41.0]	44.0 [42.3–45.8]	4.5	<0.0001
**40–59 yo**	613	38.5 [36.9–40.3]	43.8 [41.6–46.1]	5.3	<0.0001
**60+yo**	232	46.6 [43.5–49.8]	47.0 [43.4–50.8]	0.4	0.9

* *p* value calculated using the McNemar chi-square for matched pairs. **p value calculated using the Wilcoxon signed rank test for matched pairs. ^§^Increases in prevalence of HI titres≥1∶40 and≥1∶80 between inclusion and follow-up indicated as “Δ≥1∶40” and “Δ≥1∶80”, respectively. Increase in geometric mean titres (GMTs) between inclusion and follow-up indicated as “ΔGMT.

### Comparison with prepandemic data (n = 841)

In order to compare seroprevalence between the pre- and post-pandemic periods, 841 samples collected from blood donors in 2008 in Vientiane Capital were matched for age and sex with the participants in our cohort. Prevalence and GMT values for these blood donors were high (HI≥1∶80 = 18.8%, 95% CI [16.2, 21.4]; GMT = 45, 95% CI [43.4, 46.7]). In the prepandemic population, the prevalence in the 17–19 age group (HI≥1∶80 = 18.7%, 95% CI [15.0, 22.5]) was no higher than it was in the 20–58 group (HI≥1∶80 = 18.9%, 95% [CI 15.1, 22.6]). A significant increase between 2008 and 2009 could still be detected in the 17–19 yo group (*p* = 0.01) with a threshold of 1∶80.

### Serological changes between inclusion and follow-up in nonvaccinated participants (n = 2,810)

**Figure 3:**
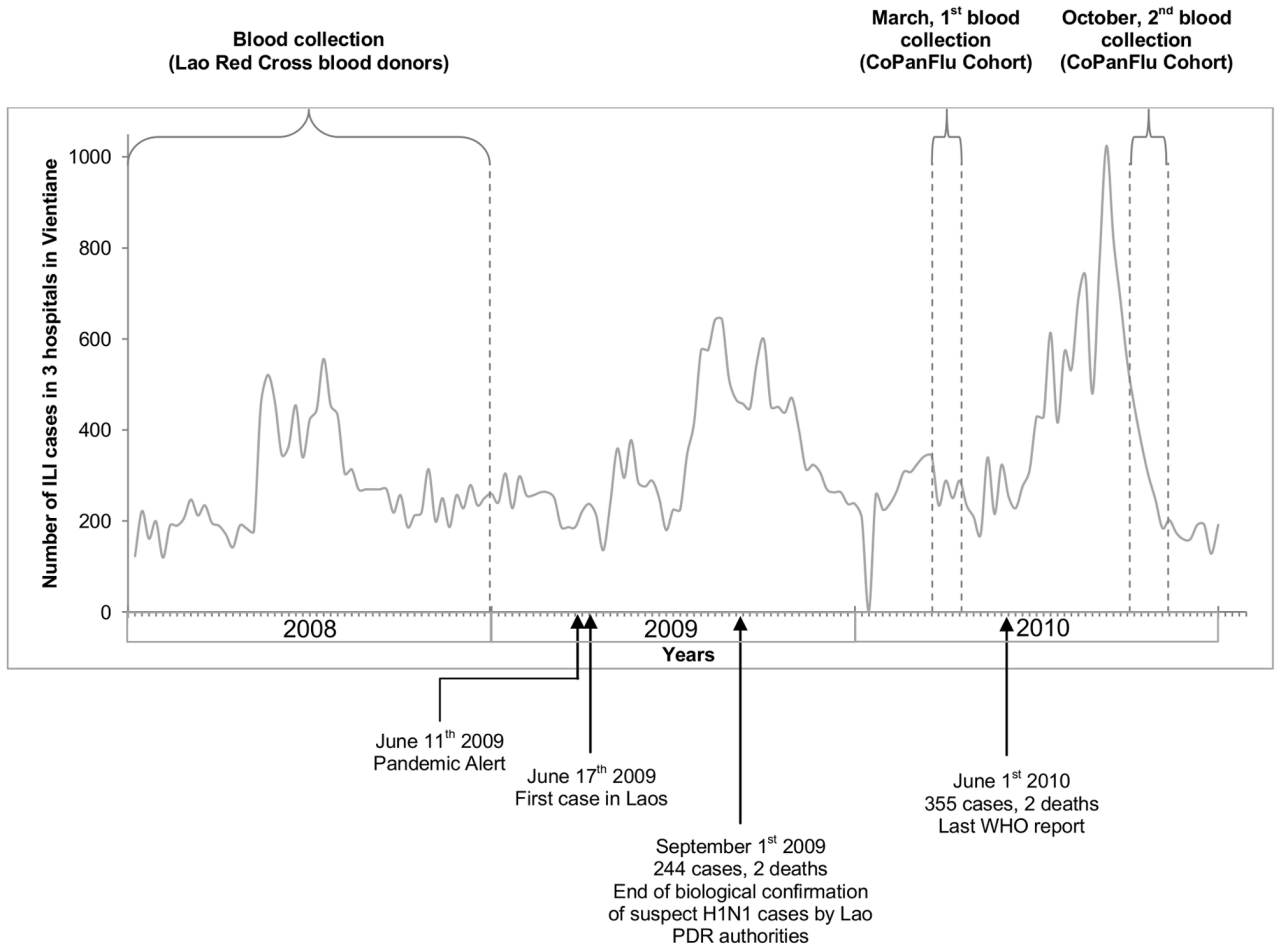
Seasonal influenza-like illness activity and chronology of the pandemic events and CoPanFlu programme.

The period between the collection of inclusion (March 2010) and follow-up (October 2010) samples within the CoPanFlu Laos cohort encompassed the second epidemiological episode of 2009 A(H1N1) infections in Laos (see Fig. 3).

The overall analysis of the nonvaccinated participants (*n* = 2,810) revealed a significant increase in seroprevalence at the thresholds of 1∶40 ((Δ≥1∶40 = 3.4, *p* = 0.0018; Table 3a) and 1∶80 (Δ≥1∶80 = 13.4, *p*<0.0001; Table 3b), and the level of HI antibodies was higher in October than it had been in March 2010 (ΔGMT = 7.5, *p*<0.0001; Table 3c). All estimates of the attack rate (*i.e.*, Δ≥1∶40, Δ≥1∶80, seroconversion rate (SCR), Tables 3 and 4) yielded the highest numbers in the 0–19 yo age group, and the lowest in the over 60 yo group.

**Table 4:** Seroconversion rates between March and October 2010 in 2,810 nonvaccinated participants.

	Seroconversion rate in nonvaccinated participants		
	Individuals with inclusion HI titre<1∶40	Individuals with inclusion HI titre≥1∶40	All groups
**All age groups**	30.3% [27.3–33.5]	6.7% [5.7–8.0]	14.3% [13.0–15.6]
**00–19 yo**	44.2% [38.5–50.0]	9.8% [7.8–12.2]	19.8% [17.4–22.4]
**20–39 yo**	24.1% [19.5–29.1]	4.3% [2.8–6.2]	11.1% [9.1–13.3]
**40–59 yo**	23.5% [18.0–29.7]	6.3% [4.1–9.2]	12.4% [9.9–15.3]
**60+yo**	17.2% [8.6–29.4]	2.9% [0.9–6.6]	6.5% [3.7–10.4]

The overall SCR amongst nonvaccinated individuals was 14.3% (95% CI [13.0, 15.6]), with a significant difference (*p*<0.0001) between participants under 20 yo (19.8%, 95% CI [17.4, 22.4]) and over 60 yo (6.5%, 95% CI [3.7, 10.4]). In the 20–59 yo group, 11.6% (95% CI [10.1, 13.3] seroconverted – a significantly higher proportion than in the over 60 yo group (*p* = 0.02) and a significantly lower one than in the under 20 yo group (*p*<0.0001).

**Figure 4:**
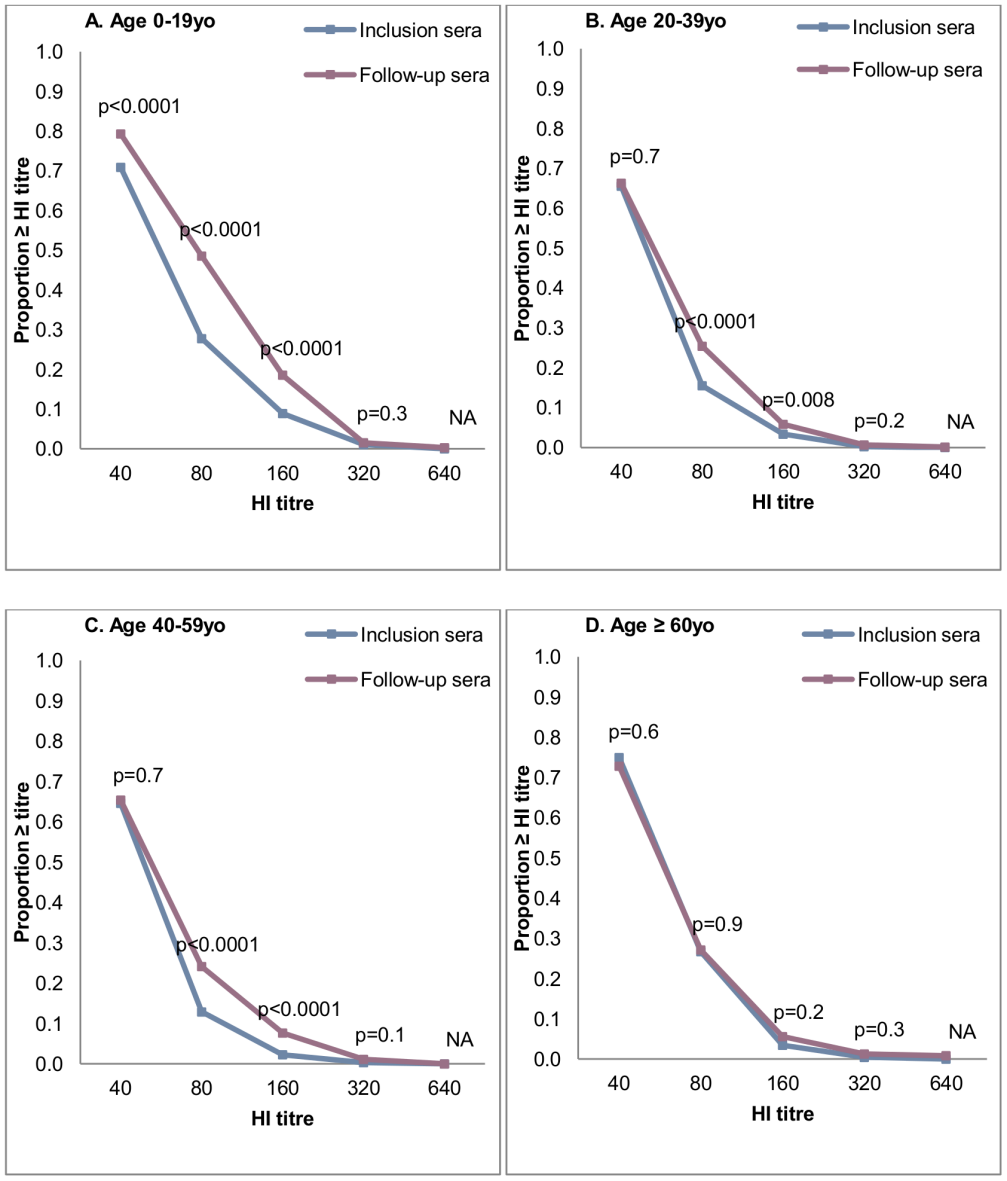
Distribution of samples at inclusion according to HI titre and age group. *p* value calculated using the McNemar chi-square for matched pairs.

Figure 4 shows the proportions of the samples at inclusion and follow-up that were equal to or above each titre level for the 0–19 yo, 20–39 yo, 40–59 yo and≥60 yo age groups. Participants under 20 yo showed significant differences between inclusion and follow-up for all titre levels between 1∶40 and 1∶160. Intermediate age groups showed significant differences for titres 1∶80 and 1∶160. No difference was observed at any titre level in individuals over 60 yo.

### Determinants of seroconversion in nonvaccinated participants (n = 2,810)

The data we had collected on the demographics, work environment, general health status, history of ILIs and other chronic and acute diseases, history of vaccination, living conditions and household environment of participants living in the three different areas of Vientiane Capital (central zone, inner urbanized belt and outer urbanized belt), were analyzed using the GEE model to identify factors associated with seroconversion.

After adjustment and tests for interactions, the final GEE multivariate model identified four independent determinants for seroconversion (see Table 5):

**Table 5:** Determinants of seroconversion between March 2010 and October 2010 in nonvaccinated participants.

Determinants of seroconversion (*n* = 2,810)			
	Odds ratio	95% CI	*p* value
**Age**			
00–19 yo	3.8	[1.9, 7.8]	0.0002
20–39 yo	1.5	[0.7, 3.1]	0.3
40–59 yo	1.6	[0.8, 3.3]	0.2
60+yo	ref		
**HI at inclusion**			
1∶10	76.5	[27.1, 215.8]	<.0001
1∶20	27.8	[10.9, 70.5]	<.0001
1∶40	8.1	[3.3, 20.4]	<.0001
≥1∶80	ref		
No. seroconverted individuals in the household	3.3	[2.8, 3.9]	<.0001
Size of the household*	0.8	[0.8, 0.9]	<.0001

Generalized estimating equation multivariate model taking the cluster effect into account. *Size of the household included in the model as a discrete variable.

(*i*) Youth(<20 yo), was found to be a determinant, with an odds ratio (OR) of 3.8, 95% CI [1.9, 7.8] (*p* = 0.0002), taking age≥60 yo as a reference;

(*ii*) An HI titre at inclusion<1∶80 was a risk factor, with a dose-effect relationship (OR ranging from 76.5, 95% CI [27.1, 215.8], for a titre at inclusion equal to 1∶10, to 8.1, 95% CI [3.3, 20.4] for a titre of 1∶40);

(*iii*) The presence in the household of one or several seroconverted individuals was also a risk factor (OR = 3.3, 95% CI [2.8, 3.9], *p*<.0001);

(*iv*) All other things being equal, a large household was a protective factor (OR = 0.8, 95% CI [0.8, 0.9], *p*<.0001).

### Serological changes between inclusion and follow-up in participants vaccinated against 2009 A(H1N1) virus (n = 705)

**Figure 5:**
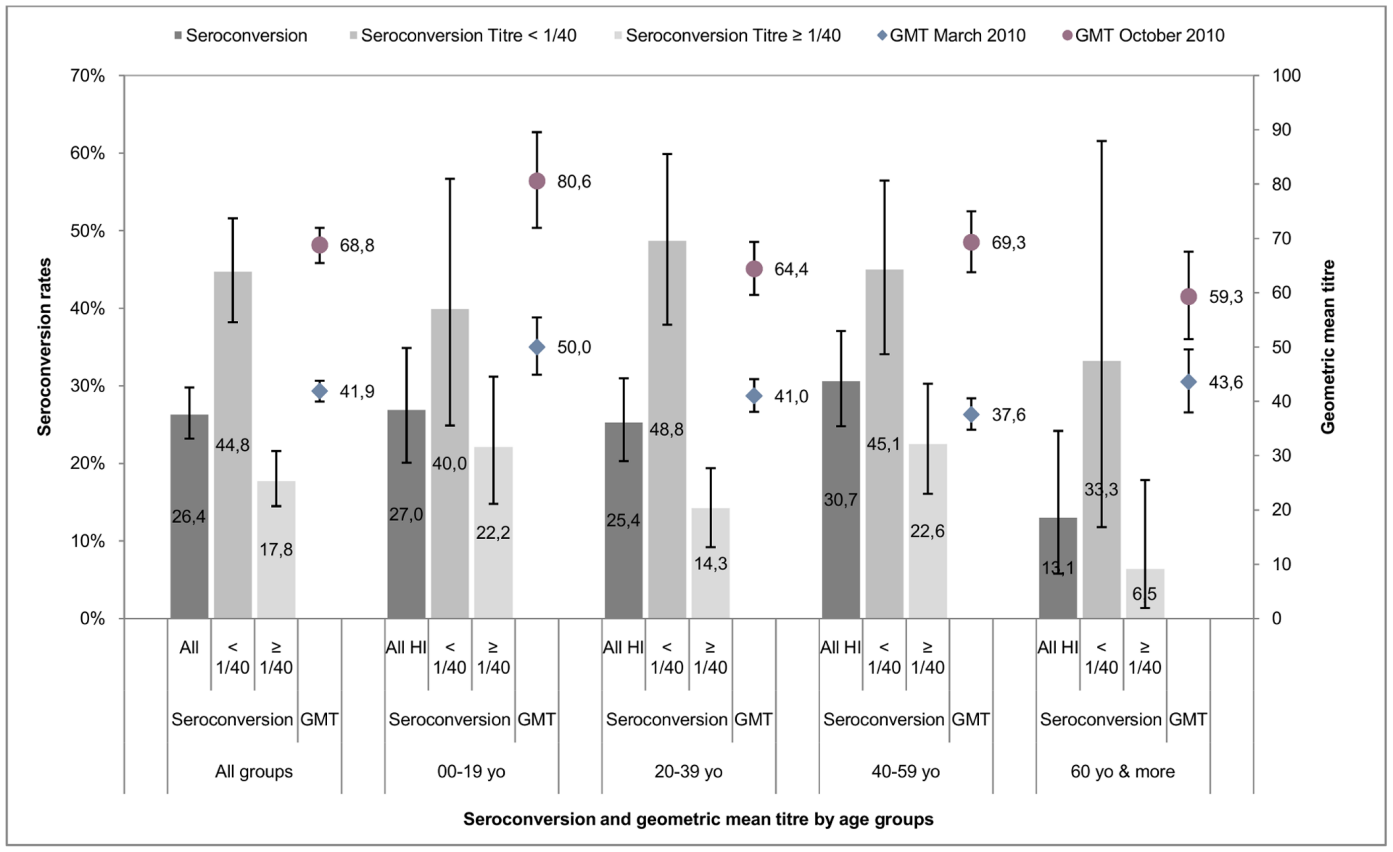
Seroconversion rates and GMT in March and October 2010 in 705 vaccinated participants. Error bars represent confidence intervals. Spv: seroprevalence. Scr: seroconversion.≥1∶40: Seroprevalence analyzed with a cutoff≥1∶40.≥1∶80: Seroprevalence analyzed with a cutoff≥1∶80.

The vaccination rate varied across age groups. Children (0–19 yo) were less likely to be vaccinated than older participants (12.6%, 95% CI [10.7, 14.6] vs. 24.3%, 95% CI [22.5, 26.1]; *p*<0.0001). The overall SCR for vaccinated participants was 26.4%, 95% CI [23.2, 29.8] (*n* = 186). Tables 6, 7 and Figure 5 show the SCRs and changes in GMT for each age group. Both SCRs and GMT increases were significantly lower in individuals over 60 yo than in the other age groups (*p* = 0.01 for SCRs and *p* = 0.03 for GMTs).

**Table 6:** GMT in March and October 2010 in 705 vaccinated participants.

GMT in vaccinated participants						
	*N*	March 2010	October 2010	Δ GMT	*p* value*	
**All age groups**	705	41.9 [40.0–43.8]	68.8 [65.5–72.0]	26.9	<0.0001	
**00–19 yo**	148	50.0 [44.9–55.5]	80.6 [72.0–89.6]	30.6	<0.0001	
**20–39 yo**	268	41.0 [38.1–44.1]	64.4 [59.6–69.4]	23.4	<0.0001	
**40–59 yo**	228	37.6 [34.8–40.6]	69.3 [63.8–75.0]	31.7	<0.0001	
**60+yo**	61	43.6 [38.0–49.6]	59.3 [51.5–67.6]	15.7	0.0003	

* *p* value calculated using the Wilcoxon signed rank test for matched pairs.

**Table 7:** Seroconversion rate between March and October 2010 in 705 vaccinated participants.

	Seroconversion in vaccinated participants		
	Individuals with inclusion HI titre<1∶40	Individuals with inclusion HI titre≥1∶40	All groups
**All age groups**	44.8% [38.2–51.6]	17.8% [14.5–21.6]	26.4% [23.2–29.8]
**00–19 yo**	40.0% [24.9–56.7]	22.2% [14.8–31.2]	27.0% [20.1–34.9]
**20–39 yo**	48.8% [37.9–59.9]	14.3% [9.2–19.4]	24.5% [20.3–31.0]
**40–59 yo**	45.1% [34.1–56.5]	22.6% [16.1–30.3]	30.7% [24.8–37.1]
**60+yo**	33.3% [11.8–61.6]	6.5% [1.4–17.9]	13.1% [5.8–24.2]

## Discussion

Deciphering the epidemiological characteristics and impact of the 2009 influenza pandemic is an important public health objective. It will lead to a better understanding of the public health implications of influenza outbreaks, improvements in epidemiological modelling, and more efficient management of future influenza epidemics. Sero-epidemiological studies are the cornerstone of strategies for estimating influenza attack rates in the general population, as there are too many obstacles (*e.g*., presence of asymptomatic or subclinical cases, wide disparities in healthcare seeking behaviour) for clinical reporting systems to efficiently account for all infected cases.

So far, most of the studies designed to record pandemic 2009 A(H1N1) attack rates have been based on cross-sectional serological analyses. A clear limitation to the comparison of results obtained in independent series is the significant variability in the production of results, mostly due to differences in technical protocols (*e.g.*, antigen preparation, pre-analytic processing of samples, choice of red blood cells) and endpoint analysis methods [14]–[16]. The gold standard for providing sero-epidemiological estimates of the attack rate is individual seroconversion studies, in which blood samples are obtained before and after the epidemiological episode under investigation, and which thus rely on pre-existing cohorts. For obvious reasons, this constraint has limited the number of studies based on individual seroconversion that have been conducted on the 2009 A(H1N1) pandemic [7], [8], [17]–[24], some of these studies being dedicated to specific populations such as HIV patients or hospital staff.

Consequently, conducting general population-based studies using individual paired samples collected before and after the different waves of infection of the 2009 A(H1N1) variant is crucial if we are to properly assess pandemic infection rates [25].

### Seasonal patterns

Seasonal patterns in influenza activity in human populations in East and Southeast Asia exist, but are not uniform across the region. Periods of moderate to high activity typically last longer in tropical and subtropical regions than in temperate ones, and occur more frequently than once a year [26]. Both Cambodia and Thailand seem to experience an influenza season between July and December, with a peak during the rainy season [27], [28]. Data from the NCLE point to a similar trend in Laos, with year-round ILI activity and a peak during the rainy season (May-October) (unpublished results). This is illustrated in Figure 3, which shows the ILI cases reported to the NCLE by three hospitals in Vientiane Capital in 2008–2010, together with the chronology of the main events regarding the pandemic influenza and the CoPanFlu Laos study.

### Haemagglutination Inhibition Assay

The HI analyses in our study indicating the proportions of the samples at inclusion and follow-up that were equal to or above each titre level in each age group suggest that titre of 1∶80 may represent the most relevant threshold for the specific diagnosis of recent 2009 A(H1N1) infections (Fig. 4), consistent with previous observations [8], [12]. This is further supported by the fact that the Δ≥1∶80 yielded an estimate of attack rate similar to that obtained from SCRs and illustrated by comparing the HI titres in each age group at inclusion and follow-up. There is therefore a line of evidence that the antibody level≥1∶80 was quite specific to pandemic H1N1 infection. However, in a significant proportion of the prepandemic samples, antibodies cross-reacting with the 2009 A(H1N1) antigen could be detected at titres of≥1∶40. Given the cohort's age range (17–58 yo), this finding probably denoted cross-reactivity with recent seasonal flu strains, as observed in younger age groups in developed countries [16], [29]. The prevalence of a pandemic HI titre≥1∶80 was also substantial in our prepandemic samples, and was more unexpected, given the results of previous analyses using the same technique [8], [12]. The most probable explanation is that that plasma samples were obtained from the blood donors, whereas we collected serum samples from our cohort. It could also be explained, however, by specific previous exposure to influenza strains within the Laotian population or, more specifically, within the control group, leading to a matching bias.

### First round of blood collection, March 2010

The study began in March 2010, after the first wave of 2009 A(H1N1) virus in Laos. The inclusion-sampling period took place 5 months after the previous 2009 ILIs peak (see Fig. 3). Since it has been established that the 2009 A(H1N1) antibody titre could decrease rapidly in certain individuals once the infectious episode was over [7], [12], the 2009 A(H1N1) seroprevalence data at inclusion may underestimate the actual number of previous infections.

Serological analysis provided a picture consistent with what has been reported in other countries: whichever HI threshold was considered, the highest prevalence levels and HI titres were identified in (*i*) individuals under 20 yo, reflecting the fact that this age group was the primary target of the pandemic virus [7], [8], [12], [17]–[19], [30]–[38], and (*ii*) the elderly. Elderly individuals were paradoxically poorly impacted by the first wave, most probably because of pre-existing cross-reactive antibodies. They may have been infected with Spanish flu-related A(H1N1) strains prior to the major antigenic drift of A(H1N1) viruses that occurred at the end of the 1940s [39]. These antibodies had been detected in individuals over 60 yo in most previous sero-epidemiological studies [7], [8], [17], [30], [32], [38], [40]–[52], with the exception of three studies in China [53], Taiwan [19], and Singapore [54], where none of the participants over 60 yo was seropositive for pandemic 2009 A(H1N1) antibodies.

It has been suggested that iterative vaccination against seasonal influenza may provide another plausible explanation for the presence of cross-reactive antibodies in the elderly [47], [53], [54], because this age group benefits from the highest vaccine coverage in developed countries. The current study does not support this hypothesis, and suggests that this observed immunity is more likely due to a natural pre-exposure to influenza strains since only one of the participants over 60 yo enrolled in our study had previously been vaccinated against seasonal influenza virus, and then only once.

### Comparison with prepandemic data

Our attempt to estimate prepandemic seroprevalence by studying blood donors sampled in 2008 was not completely satisfactory. The background noise observed in the results of plasma samples made it hard to perform the relevant fine-tuned comparisons, and we were thus only able to surmise that individuals aged 17–20 years had been the major epidemiological target of the first pandemic wave. A surrogate estimate of the attack rate during the 2009 wave could be provided by analyzing the distribution of low-and high-titre antibodies. After the first wave of the pandemic, a high prevalence of antibodies was observed (68.1%), including a large proportion of low titre antibodies (47.7% of the studied population had an HI titre of 1∶40). Some of these low-titre antibodies may have been acquired during the first pandemic wave, but were indistinguishable from cross-reactive antibodies acquired over time against a cocktail of variants of influenza viruses that can differ from country to country, reflecting different regional epidemiological histories. It is worth noting that, in the case of Laos, at the time of inclusion, (*i.e*., after the 2009 wave), the distribution of HI titre at 1∶40 across the age groups was such that prevalence was lowest in individuals younger than 20 yo, who were the major epidemiological target of the first pandemic wave.

We were able to compare the Laotian data with data collected in other population cohorts after the 2009 wave, using the same HI technical protocol and the same interpretation criteria. In Reunion Island, for instance, the prevalence of antibodies was 55% (33% with an HI titre of 1∶40) [7], and in Mali the prevalence of antibodies was 29% (13% with an HI titre of 1∶40) [8]. In both cases, the prevalence of antibodies at titres >1∶40 (22% in Reunion island and 16% in Mali) provided robust estimates of strict seroconversion rates (*i.e.* 20% in Reunion island and 14% in Mali). Similarly, in the case of Laos, the prevalence of antibodies with titre >1∶40 at the time of inclusion suggests that the attack rate during the 2009 wave was in the order of 20% and confirms the predominance of infection in those under the age of 20 (28%).

Different HI technical protocols implemented in different studies can potentially compromise comparisons of results with data yielded by the CoPanFlu Laos programme. Regarding the specificity of the CoPanFlu HI protocol, if we consider a seropositivity threshold of 1∶80, the most relevant one for the specific detection of 2009 A(H1N1) antibodies [12], Laotian rates can be cautiously compared with those of other countries. Estimated seroprevalence within the general population worldwide following the first 2009 wave ranged from 3% in Norway [36] to 28% in Oceania [32]. Our seroprevalence results were close to those observed in the USA [38], UK [33], and China [37], and slightly lower than those observed in Oceania [30], [32].

### Serological changes between March 2010 and October 2010

In October 2010, six months after the first blood sample collection round in the CoPanFlu cohort, a second round was conducted within the cohort. This second round, coinciding with the start of the rainy season, lasted until early November 2010. These two rounds encompassed the second epidemiological episode of 2009 A(H1N1) in Laos (see Fig. 3), the follow-up samples being obtained shortly after the influenza peak – a favourable situation for optimized antibody detection. This gave us the opportunity to analyze the serological results for paired inclusion/follow-up samples and to obtain estimates of the attack rates for each age group.

Comparisons of the serological data for the nonvaccinated participants reinforced what had been observed in the first phase: participants below 20 yo were clearly most heavily impacted by the 2009 A(H1N1) infection, with an SCR of 19%, while those over 60 yo were the least affected by the virus, with an SCR of 6.5%. The Δ≥1∶80 observed between the two periods was fully consistent with the SCR for the 0–59 yo groups, but underestimated the attack rate amongst the elderly, probably owing to the seronegation observed in this group between the two phases. By the end of the second wave, it was estimated that as many as 50% of school-aged children in Vientiane Capital had been infected. As observed elsewhere, participants<20 yo were and remained the key target of this pandemic during the second wave [17], [36], [40].

The impact of the second 2009 A(H1N1) wave has been less extensively explored and, to our knowledge, no studies featuring paired serum samples have so far been published on the subject. Only three studies based on matched data have so far assessed the impact of Wave 2 of the 2009 A(H1N1) virus, in Canada (Ontario) [17], the UK (England) [40], and Norway [36]. The cumulative incidence of infection we found was lower than the attack rates reported in these three studies (26.3% in England, 27.6% in Ontario and 43.3% in Norway).

### Determinants of seroconversion

Our study included more than one hundred variables describing the situation of each participant in terms of his or her geographical location, demographics, work environment, general health status, history of ILIs and other chronic and acute diseases, history of vaccination, living conditions and household environment. The final multivariate GEE model, taking household clustering into account, identified only four independent determinants for seroconversion. As expected, youth was found to be a strong determinant of seroconversion, with an OR of 3.8, 95% CI [1.9, 7.8]. Our analysis also suggested that those with lower baseline titres had significantly higher infection rates, with a clear dose-effect relationship, with ORs ranging from 76.5 (95% CI [27.1, 215.8]), for those with an HI at inclusion of 1∶10, to 8.1 (95% CI [3.3, 20.4]), for those with an HI of 1∶40. The two other epidemiological determinants, corroborated in the literature, were having another household member with a level of antibody titre≥80, which was associated with a higher likelihood of immunity [23], and belonging to a large household, which reduced the likelihood of being infected. This last result may seem counterintuitive but is reminiscent of Cauchemez *et al.* 's finding [55], [56]. These authors suggested that a “time-sharing” mechanism is at work for infection within the household: the duration of contact between two members of a household can basically be divided by the total number of household members. Thus, the more crowded the household is, the less face-to-face time one member spends with another, thereby decreasing exposure times between individuals. However, as the authors mentioned, the sociological, environmental, and biological mechanisms behind the relationship between secondary attack rates and household size have yet to be elucidated.

A second explanation may be related to the specific structure of Laotian households, which are far larger than the nuclear family. They include members of the extended family, some of them elderly. In Southeast Asia, the elderly fulfil a social function within the household, for since they can no longer offer financial support, land or other material goods, they provide childcare assistance and perform household duties [57]. From this perspective, in large households, infected individuals are likely to be taken care of by elderly family members, and transmission in this particular instance may have occurred less frequently because these elderly family members were less at risk of infection for this particular virus.

## Conclusion

Our study suggests that the profile of the 2009 A(H1N1) pandemic in Laos was similar to that observed in other countries, regardless of differences in environmental conditions, overall socio-economic status or climatic specificities, with strong independent epidemiological determinants characterizing this event. Moreover, we detected pre-existing cross-reacting antibodies in individuals over 60 yo, which could not have originated from earlier multiple vaccination as has been suggested elsewhere. In a developing country such as Laos, laboratory confirmation cannot be performed for most cases, and healthcare seeking is far from systematic. For this kind of particular event, using serological cohorts is therefore one of the best ways of estimating infection rates, and our study highlights the value of serological data as a complement to clinical surveillance.
